# Renal Involvement in Hereditary Transthyretin Amyloidosis: An Italian Single-Centre Experience

**DOI:** 10.3390/brainsci11080980

**Published:** 2021-07-24

**Authors:** Pietro Manuel Ferraro, Viola D’Ambrosio, Andrea Di Paolantonio, Valeria Guglielmino, Paolo Calabresi, Mario Sabatelli, Marco Luigetti

**Affiliations:** 1 Fondazione Policlinico Universitario A. Gemelli IRCCS, UOC Nefrologia, 00168 Rome, Italy; pietromanuel.ferraro@unicatt.it (P.M.F.); viola.dambrosio01@icatt.it (V.D.); 2 Università Cattolica del Sacro Cuore, Sede di Roma, Largo A. Gemelli 8, 00168 Roma, Italy; andrea.dp89@gmail.com (A.D.P.); guglielmino.valeria@gmail.com (V.G.); paolo.calabresi@policlinicogemelli.it (P.C.); mario.sabatelli@unicatt.it (M.S.); 3 Fondazione Policlinico Universitario A. Gemelli IRCCS, UOC Neurologia, 00168 Rome, Italy; 4 Centro Clinico NEMO Adulti, 00168 Rome, Italy

**Keywords:** TTR, amyloid, nephropathy, proteinuria, neuropathy, Sudoscan

## Abstract

Objective: Hereditary transthyretin amyloidosis (ATTRv) represents a diagnostic challenge considering the great variability of clinical presentation and multiorgan involvement. In the present study, we report the prevalence of kidney involvement and kidney function over time in a cohort of ATTRv patients with different transthyretin gene mutations. Patients and Methods: For this study, we systematically collected data from all patients with a diagnosis of ATTRv followed at the Neurology Unit of Fondazione Policlinico Universitario A. Gemelli IRCCS. Kidney involvement was defined as presence of estimated glomerular filtration rate (eGFR) < 60 mL/min/1.73 m^2^ obtained with CKD-EPI equation, abnormal urinary protein excretion (UPE) (>150 mg/24 h) and/or albuminuria > 30 mg/24 h (or mg/g creatinine). The analysis included data from 46 patients with 122 measurements of serum creatinine. Results: Among the 46 patients included in the analysis, kidney involvement was present in 37%, with 15% showing reduced eGFR and 22% abnormal UPE (63% of patients with available UPE data). No single predictor was associated with either eGFR values or its slope over time. Conclusions: Kidney involvement is quite common in patients with ATTRv regardless of the underlying genetic variant. In particular, abnormal UPE appears to be a common feature of the disease.

## 1. Introduction

The term “amyloidosis” encompasses different disease entities deriving from conformational changes in native, soluble proteins that misfold and aggregate extracellularly into insoluble, highly ordered fibrils, leading to dysfunction of different organ and tissue [1]. Kidney is a potential target organ [2]. Proteins capable of producing amyloid deposits involving the kidney include immunoglobulin lights chains (AL), immunoglobulin heavy chains (AH), serum amyloid A (AA), fibrinogen Aα-chain (AFib), lysozyme (Alys), apolipoprotein AI (AApoAI), apolipoprotein AII (AApoAII), Leukocyte Chemotactic Factor-2 (ALECT2), beta-2 microglobulin (Aβ2M, dialysis-related amyloidosis), and transthyretin (ATTR) [1,2]. While kidney involvement is well-known and common in AL and AA forms of amyloidosis, very few data are available on kidney involvement in hereditary transthyretin amyloidosis (ATTRv), especially in late-onset patients from non-endemic areas [1,2,3].

ATTRv is a rare disease due to mutations in the gene encoding TTR and is characterized by multisystem extracellular deposition of amyloid [3]. More than 120 TTR variants have been described as a cause of ATTRv, the most frequent being the Val30Met mutation [4].

ATTRv amyloidosis represents a diagnostic challenge considering the great variability in clinical presentation and multiorgan involvement. Generally, patients present with polyneuropathy, but clinicians should also consider the frequent cardiac, ocular and gastro-intestinal impairment [3,4,5,6]. However, the pattern of clinical impairment may vary according to the geographic areas. In endemic areas, namely Portugal, patients present with early-onset (third to fourth decade) ATTRv and deteriorate quickly because of autonomic dysfunction and rapid progression of the sensory-motor deficit [7]. Conversely, in non-endemic areas, many patients present with late-onset ATTRv and the polyneuropathy (affecting predominantly the large nerve fibres) progresses slowly, often with cardiac involvement but with less autonomic dysfunction [8].

Kidney involvement in ATTRv, usually presenting as nephrotic syndrome and/or progressive renal failure, has been reported in about one-third of Portuguese/early-onset Val30Met patients (especially in those with a later onset), and in only 6% of sporadic/late-onset ATTRv cases [3,5,9].

Current therapeutic options include TTR tetramer stabilizers (diflunisal and tafamidis), agents designed to stabilize the normal circulating form of TTR, and, hence, to prevent the protein from dissociating and undergoing the conformational changes that lead to its aggregation as amyloid, and gene-silencers (patisiran and inotersen), able to decrease both variant and wild-type TTR hepatic production by targeting its mRNA [10,11,12,13]. Therapies increase the survival of patients, slowing the progression of polyneuropathy and/or cardiomyopathy [10,11,12,13]. No therapy is approved for ATTRv nephropathy, even if some effect of tafamidis in reducing proteinuria has been described [14]. A more thorough knowledge of the frequency and characteristics of kidney involvement in patients with ATTRv is thus needed.

In the present study, we report the prevalence of kidney involvement and trajectories of kidney function over time in a cohort of ATTRv patients with different mutations coming from Italy, a non-endemic region.

## 2. Materials and Methods

For this study, we systematically collected data from all patients with a diagnosis of ATTRv followed at the Neurology Unit of Fondazione Policlinico Universitario A. Gemelli IRCCS. Information was collected regarding the genetic variant, age at onset, age at examination, gender, presence of a polyneuropathy and/or a heart disease, Sudoscan measurements, as well as all the available data of urinary protein excretion and serum creatinine for each patient. Presence of polyneuropathy was defined based on nerve conduction studies, as previously described [15,16]. Sudoscan, a fairly recent technique that provides a quick, non-invasive, and quantitative assessment of the sudomotor function, was tested as previously reported [13,16]. This tool is a reliable marker of disease progression in late onset ATTRv amyloidosis patients, and it can detect nerve small fibres involvement in this setting [16].

Heart disease was defined in the presence of a NYHA class > 1. All patients were genotyped; for statistical analysis patients were classified into the following genetic categories: “Val30Met”, “Phe64Leu” and “Other”. Estimated glomerular filtration rate (eGFR) was obtained with the CKD-EPI equation. Kidney involvement was defined as any eGFR < 60 mL/min/1.73 m^2^, abnormal urinary protein excretion (UPE) (>30 mg/24 h [or mg/g creatinine] for albumin, >150 mg/24 h for total protein) or both. An analysis of predictors of both eGFR values at any time and of eGFR slope (longitudinal analysis) was performed in patients with at least two measurements of serum creatinine over time by random-intercept mixed models, with eGFR modelled as the continuous dependent variable and potential clinical and instrumental predictors as the independent variables. To determine whether predictors of interest were associated with the slope of eGFR, interaction terms for each predictor and time were included in the models. Time was modelled as a continuous variable, measuring months lapsed from the first measurement of serum creatinine.

A two-tailed *p*-value < 0.05 was considered as statistically significant. All statistical analyses were performed with Stata version 15.1 (Stata Statistical Software: Release 15. College Station, TX, USA: StataCorp LLC). This study was approved by the Ethics Committee of Fondazione Policlinico Universitario A. Gemelli IRCCS (ethic code number ID1493). All the patients who participated to the study gave an informed consent. This study was carried out according to the Declaration of Helsinki.

## 3. Results

Overall, 46 patients had at least one measurement of serum creatinine available (122 total measurements). Of those 46 patients, 11 (24%) had 1 measurement, 12 (26%) had 2 measurements, 10 (22%) had 3 measurements, 9 (20%) had 4 measurements, 3 (7%) had 5 measurements and 1 (2%) had 6 measurements. Among those with at least 2 measurements, median follow-up time was 56 months (minimum 3, maximum 193 months). Sixteen patients (35%) had at least one measurement of urinary protein excretion. Baseline characteristics of the enrolled patients are reported in Table 1.

The majority of patients had the Val30Met variant (*n* = 18, 39%) or the Phe64Leu variant (*n* = 19, 41%); the remaining patients had the Glu89Gln variant (*n* = 4, 9%), or other variants (*n* = 5, 11%). Overall, the presence of kidney involvement was high: low eGFR during any time of follow-up was present in 7 patients (15%), abnormal UPE in 10 (22% of the sample, 63% of those with available urinary data); when considering a combination of low eGFR and/or abnormal UPE, 17 patients (37%) had kidney involvement. At baseline, only two patients presented low eGFR (one Val30Met and one Phe64Leu); during follow-up, an additional 5 cases developed low eGFR (three Val30Met; one Glu89Gln; one Ala109Ser). Conversely, abnormal UPE was found in 4 Val30Met patients, 3 Phe64Leu and 3 with other single mutations (Glu89Gln; Ile88Leu; Thr59Lys). Indeed, in one Val30Met patient a nephrotic syndrome represented the first sign of ATTRv.

In univariate analyses, eGFR values at any time were inversely correlated with female sex (−11.00 mL/min/1.73 m^2^, 95% CI −21.77, −0.24) and age (for each 1 year −0.60 mL/min/1.73 m^2^, 95% CI −1.18, −0.03). However, in multivariate analysis eGFR was significantly associated only with age (for each 1 year −0.72 mL/min/1.73 m^2^, 95% CI −1.17, −0.28) (Table 2).

**Figure 1:**
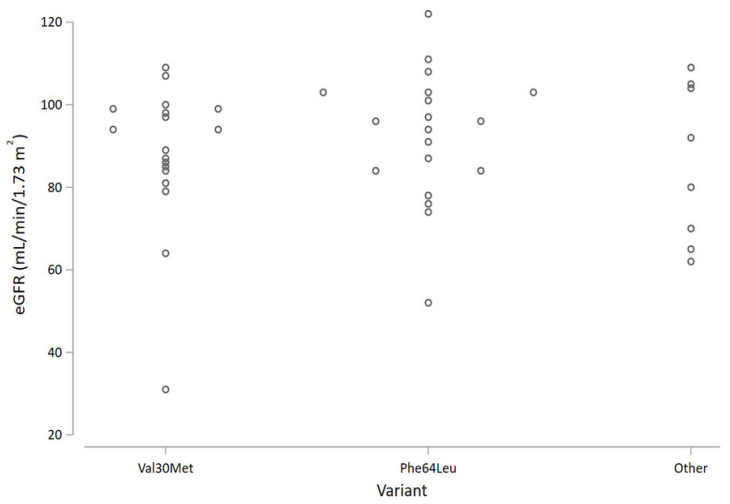
Strip plot of eGFR by variant.

Focusing on the genetic variant, patients with the Phe64Leu mutation had nominally higher average eGFR values compared with the Val30Met (Figure 1); however, the difference was not statistically significant.

The analysis of eGFR slopes, performed on the 35 patients with at least 2 creatinine measurements (111 total measurements), revealed that the average change in eGFR was −1.00 mL/min/1.73 m^2^ per year (95% confidence interval [CI] −1.63, −0.37). No predictor was significantly associated with the slope of eGFR over time (Table 3).

## 4. Discussion

Kidney involvement is well-known in early-onset ATTRv, and it generally occurs in one third of patients [3,5,10]. Conversely, few data are available on late onset ATTRv where kidney involvement is thought to be rare, and related to specific mutations [3,5,9].

In our cohort of late-onset ATTRv, we reported the presence of kidney involvement in more than one third of patients, a much higher figure compared with that of the general population, suggesting that kidney involvement might be quite common and underestimated in ATTRv patients.

Considering different mutations, we did not find significant differences at baseline. However, kidney involvement seems to be more frequent in Val30Met, being present in more than one third of cases, similar to what is described in early-onset Val30Met [5]. On the other hand, the rate of loss of eGFR over time does not differ significantly across TTR genetic variants. 

Kidney disease may be one of the presenting manifestations in early-onset Val30Met [5]. Conversely, in late-onset ATTRv, kidney involvement at diagnosis is relatively rare, being found only in one patient of our cohort. However, we noticed that occurrence of kidney disease increases during follow-up, if properly investigated. We strongly suggest adding serum creatinine (and possibly cystatin C, especially in patients with muscular atrophy) and UPE in the routine diagnostic work-up and follow-up of ATTRv patients [3]. Given the difficulties concerning the 24 h urine collection in these patients, spot-urine protein-to-creatinine ratio (PCR) is an accurate, valid, and reliable method to estimate UPE, as recommended by KDIGO (Kidney Disease: Improving Global Outcomes) guidelines, especially in a screening setting. In particular, the finding of a strikingly high prevalence (63%) of abnormal UPE among patients with available urinary data suggests that this might be a feature of ATTRv, and that kidney involvement in our cohort would have been even more common if urinary data had been available in a larger number of patients. Given the role of abnormal UPE as an independent risk factor for progression of kidney disease, urinary investigations should be systematically sought in ATTRv patients. Drugs that effectively reduce UPE, such as inhibitors of the renin-angiotensin-aldosterone system, should be considered in proteinuric patients, although with careful monitoring of blood pressure.

Our study has several strengths, including the large number of patients for a rare disease, the longitudinal design with multiple measurements of serum creatinine for each patient, the presence of different genetic variants, and the statistical analysis performed with a gold-standard approach, useful for longitudinal data. Our study also has some limitations, including the observational design, the relatively small number of measurements for some variants, and the lack of UPE for most patients.

## 5. Conclusions

In conclusion, ATTRv confirms to be a multisystemic disease that can frequently also involve the kidney, even in late onset cases. Serum and urinary biomarkers of renal damage (serum creatinine, cystatin C, azotemia, UPE, urinalysis) should be investigated early, both in diagnostic work-up, in follow-up, and in clinical trials.

Future studies on a larger ATTRv cohort and on international registries, such as THAOS, will help clarify the real prevalence of renal disease in ATTRv. Furthermore, the role of therapies (including stabilizers and gene-silencers) on kidney function should be evaluated, as well as the role of prolonged survival of ATTRv patients in the occurrence of kidney disease, thus strengthening the role of the nephrologist in this disease.

## Figures and Tables

**Table 1:** Baseline characteristics of the enrolled patients.

**Age (Years), Mean (SD)**	66.3 (7.9)
**Age at Onset (Years), Mean (SD)**	61.8 (9.0)
**Female Sex, n (%)**	12 (26%)
**Genetic Variant** **Ala109Ser** **Ala120Ser** **Glu89Gln** **Ile88Leu** **Phe64Leu** **Thr59Lys** **Val30Met**	1 (2%) 2 (4%) 4 (9%) 1 (2%) 19 (41%) 1 (2%) 18 (39%)
**Intraventricular Septal Thickness (mm), Mean (SD)**	14.9 (4.0)
**Abnormal Sudoscan, n (%)**	24 (73%)
**Heart Disease, n (%)**	14 (33%) *
**Neuropathy, n (%)**	43 (96%)
**Abnormal Proteinuria, n (%)**	10 (63%)
**eGFR (mL/min/1.73 m^2^), Mean (SD)**	89.3 (17.0)
**Low eGFR, n (%)**	2 (4%)

* Two of these patients also required a pacemaker for heart rhythm abnormalities.

**Table 2:** Predictors of eGFR at any time.

	Univariate Model		Multivariate Model	
Factor	Beta (95% CI)	*p*-Value	Beta (95% CI)	*p*-Value
Age	−0.60 (−1.18, −0.03)	0.041	−0.72 (−1.17, −0.28)	0.001
Female sex	−11.00 (−21.77, −0.24)	0.045	−1.23 (−10.94, 8.48)	0.803
Variant (Val30Met = Ref) Phe64Leu Other	6.18 (−4.53, 16.90) −3.04 (−16.19, 10.12)	0.258 0.651	4.62 (−5.14, 14.37) −11.85 (−24.63, 0.93)	0.354 0.069
Heart disease	−8.82 (−19.36, 1.73)	0.101	−4.27 (−16.77, 8.23)	0.503
Neuropathy	−7.94 (−30.50, 14.62)	0.491	−1.29 (−19.35, 16.77)	0.889
Abnormal Sudoscan	−6.43 (−15.60, 2.75)	0.170	−0.66 (−12.36, 11.03)	0.911
Intraventricular septal thickness	−0.96 (−2.29, 0.36)	0.153	0.44 (−0.94, 1.83)	0.531

**Table 3:** Predictors of slope of eGFR.

Factor	*p*-Value
Age	0.225
Female sex	0.359
Variant (Val30Met = Ref) Phe64Leu Other	0.464 0.658
Heart disease	0.147
Neuropathy	0.821
Abnormal Sudoscan	0.840
Intraventricular septal thickness	0.524

## Data Availability

Data available from authors.
